# Dynamic hybridization between two spleenworts, *Asplenium incisum* and *Asplenium ruprechtii* in Korea

**DOI:** 10.3389/fpls.2023.1116040

**Published:** 2023-07-05

**Authors:** Hyoung Tae Kim, Sang Hee Park, Jung Sung Kim

**Affiliations:** ^1^ Department of Ecological and Environmental System, Kyungpook National University, Sangju, Republic of Korea; ^2^ Department of Forest Science, Chungbuk National University, Cheongju, Republic of Korea

**Keywords:** *Aplenium*, hybridization, polyploidization, genome size, plastome, genotypes

## Abstract

Natural hybridization between *Asplenium incisum* and *A. ruprechtii* has been observed in Northeast Asia and its allotetraploid species, *A. castaneoviride*, was reported. However, the hybridization process between the parental species and the origin of the allotetraploid taxon remains obscure. Additionally, the systematic affinities of the recently described hybrid *A. bimixtum*, considered to have originated from the hybridization of *A. ruprechtii*, *A. trichomanes*, and *A. incisum*, is unresolved owing to its similarity to *A. castaneoviride*. The goals of this study were to (1) investigate the hybridization between *A. ruprechtii* and *A. incisum*; (2) verify the origin of *A. castaneoviride* occurring in Korea, whether it independently arose from 2x sterile hybrids; and (3) elucidate the reliability of identifying *A. bimixtum*. Three genotypes, *A. incisum*, *A. ruprechtii*, and their hybrid, were identified based on the nuclear gene *pgiC* sequence and finally divided them into six types by ploidy levels: diploid *A. incisum*, *A. ruprechtii*, and four hybrid types (diploid *A. × castaneoviride*, triploid *A. × castaneoviride*, allotetraploid *A. castaneoviride*, and *A. bimixtum*). In the analyses of plastid DNA, all hybrids had an *A. ruprechtii*-type *rbcL* gene. In addition, the four plastomes of *A. ruprechtii* and the hybrids had high pairwise sequence identities greater than 98.48%. They increased up to 99.88% when a large deletion of *A.* x *castaneoriviride* (2x) collected from Buramsan populations was ignored. Notably, this large deletion was also found in triploid *A. × castaneoviride* and allotetraploid *A. castaneoviride* in the same populations. Sequence data of the nuclear and plastid genes showed that hybridization is unidirectional, and *A. ruprechtii* is the maternal parent. The large deletion of *rpoC2-rps2* commonly found in the different ploidy hybrids of the Buramsan population suggests that the allotetraploid *A. castaneoviride* can be created independently from sterile hybrids. We assume that both polyploidization driving allopolyploidy and minority cytotype exclusion took place independently in the population, since *A castaenoviride* co-occurs with *A. ruprechtii* in small populations. Furthermore, it was also observed that an enlarged noncoding region in fern organelle (ENRIFO) of the plastome was found in the genus *Asplenium*.

## Introduction

Among the processes involved in plant evolution, polyploidization plays an important role in species formation (Stebbins, 1940). Especially in ferns, most homosporous species are likely to be derived from ancient polyploidy for their high basic chromosome number (Wagner and Wagner, 1980). Comparison of chromosome numbers shows that polyploidization affects speciation, and frequent successive polyploidization was regarded to have occurred in ferns more than angiosperms (Wood et al., 2009).

*Asplenium* Lis is a widely distributed subcosmopolitan fern consisting of more than 700 species (Lin and Viane, 2013). Species in this genus have various ploidy levels caused by the sexual reproductive ability of unreduced gametophytes, which form not only new autopolyploids such as *A. sarelii* (2x) → *A. pekinense* (4x) but also allopolyploids like *A. sarelli* (2x) + *A. tenuicaule* (2x) → *A. anogrammoides* (4x) (Liang et al., 2021). To understand the polyplodization event in the *Asplenium*, investigations using molecular data have been done and revealed a reticulate relationship among the species of this genus (Dyer et al., 2012; Chang et al., 2013; Ohlsen et al., 2014; Chang et al., 2018).

**Figure 1:**
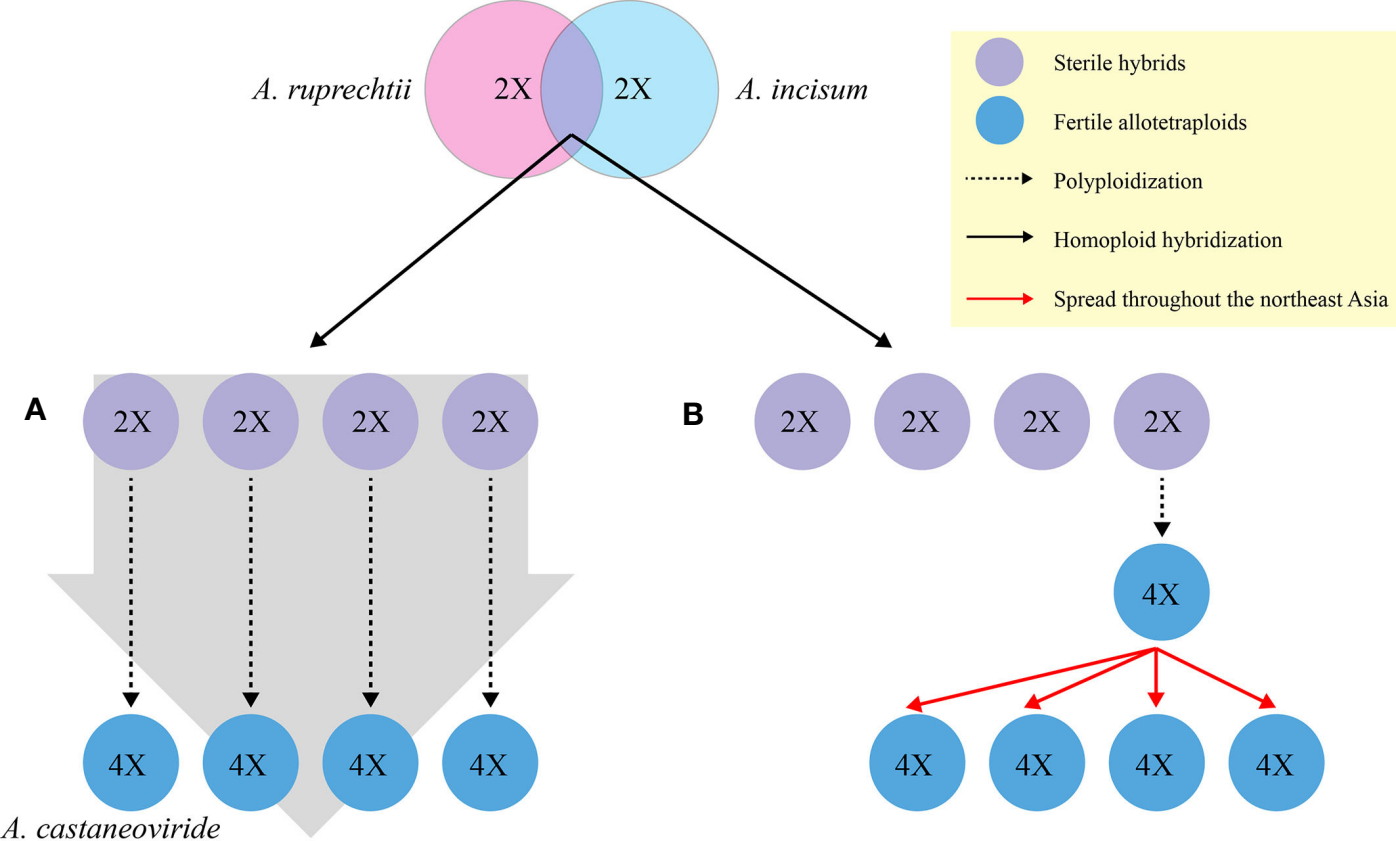
Hypotheses for the formation of allotetraploid *A castaneoviride* in Korea. **(A)** Allotetraploid *A castaneoviride* independently arises from duplication of sterile hybrids. **(B)** Allotetraploid *A castaneoviride* arises from certain sterile hybrids and spreads throughout Northeast Asia after polyploidization.

*Asplenium castaneoviride* Baker is an allotetraploid species derived from *Asplenium ruprechtii* Sa. Kurata and *Asplenium incisum* Thunb. and is distributed across China, Japan, and Korea (Lin and Viane, 2013). The fertile allotetraploid *A. castaneoviride* is close to *A. ruprechtii* and exhibits a sympatric distribution together with *A. incisum* in Korea. Based on the diploid hybrid between *A. ruprechtii* and *A. incisum* found in the natural population, we probed the origin of the allotetraploid *A. castaneovirids.* To seek answers regarding the origin of this allotetraploid species, we made the following assumptions:

If diploid hybrids are generated between *A. ruprechtii* and *A. incisum*, it can independently become a fertile allotetraploid *via* chromosome doubling (**Figure 1A**) to overcome its sterility or very low fertility, with low or identical genetic variation between progenitors and hybrids. The genetic variation in allotetraploid species may be distinguished from their progenitors if chromosome doubling occurs in certain diploid hybrids, spread with a significant divergence time throughout Northeast Asia (**Figure 1B**).

Gender-biased hybridization in ferns has captivated researchers for several years. Stein and Barrington (1990) reported an equal distribution of parental species, even though the sample size was too small to be statistically significant. In contrast, Vogel et al. (1998b) showed predominantly unidirectional hybridization between *A. septentrionale* and *A. trichomanes*, and Xiang et al. (2000) discussed how population-specific gender-biased hybridization occurs in *Dryopteris* × *triploidea*. In diploid and tetraploid hybrids of *A. ruprechtii* and *A. incisum*, it was assumed that *A. ruprechtii* was the maternal parent because it was found alongside hybrids in the field. However, this assumption is empirical and has not been experimentally proven to date. Therefore, the hybridization producing progenitors of *A. castaneoviride* remains unclear.

Another allotetraploid, *A. bimixtum* putatively derived from *A. trichomanes*, *A. ruprechtii*, and *A. incisum*, has been reported in Korea. Based on morphological features, multiple hybridization has been suggested for the development of *A. bimixtum* (Lee et al., 2015). After the first report of this hybrid, Lee et al. (2019) argued the origin of this new species based on morphological characteristics, genome size, and phylogenetic relationships. However, the origin and status of *A. bimixtum* require reconsideration since morphological features of *A. bimixtum* are almost identical to those of *A. castaneoviride*. In their cytometric study, some of the genome size data had a high standard deviation, since they ran the sample without any reference plant having the standard genome size, which generally runs together with the target sample for measuring accuracy (Lee et al., 2019). Additionally, the authors described that *A. bimixtum* has two different types of plastid genes originating from *A. ruprechtii* and *A. incisum* and suggested a multi-maternal hypothesis to explain their results. However, this is questionable because Vogel et al. (1998a) reported the maternal transmission of chloroplasts in the genus *Asplenium*, and different plant materials were used for each dataset of *rbcL* and *rps4-trnS* in their phylogenetic analysis.

The plastomes of angiosperms are normally conserved in sequence, gene content, and organization (Ruhlman and Jansen, 2014) except in certain lineages (Funk et al., 2007; McNeal et al., 2007; Cai et al., 2008; Guisinger et al., 2011; Knox, 2014). However, the plastomes of ferns have undergone more genomic mutations during evolution than those of the seed plants. Generally, genomic inversions, IR expansions/contractions, and gene duplications/deletions occur frequently throughout the fern lineages (Wolf et al., 2010; Gao et al., 2013; Grewe et al., 2013; Kim et al., 2014; Labiak and Karol, 2017). Recently, foreign DNAs, located within the plastid genome but did not match any plastome sequence, have been found in fern species at different locations (Kim and Kim, 2018; Robison et al., 2018; Lehtonen and Cardenas, 2019). These could be used as molecular markers to distinguish closely related taxa (Kim and Kim, 2020). In addition, nucleotide substitution rates for fern plastome genes are significantly higher than those for seed plant plastomes (Wolf et al., 2011). Consequently, the levels of sequence and indel divergence between intraspecific taxa in ferns are frequently higher than those of closely related intraspecific and interspecific taxa of seed plants (Kim and Kim, 2014). Owing to these dynamic variations, complete plastome sequences allow us to design molecular markers for distinguishing populations or closely related taxa in ferns.

The goals of this study were to (1) investigate the hybridization pattern between *A. ruprechtii* and *A. incisum*, (2) verify whether allotetraploid *A. castaneoviride* occurred independently originating from 2x hybrids, and (3) clarify the reliability of recognizing another allotetraploid, *A. bimixtum*, recently reported in Korea. For this purpose, we first applied two methods of genome size measurement using flow cytometry analysis and spore viability check in the manner of distinguishing a sterile hybrid with abortive spores and fertile hybrids with normal viable spores (Wagner et al., 1986) to identify the ploidy and sterility of hybrids, respectively. The complete plastome sequences of four species (*A. bimixtum*, *A. castaneoviride*, *A. incisum*, and *A. ruprechtii*) and 2x sterile hybrids between *A. incisum* and *A. ruprechtii* were compared to design a molecular marker to identify the maternal inheritance of each individual. A codominant nuclear DNA marker *pgiC* was also used to identify paternal hybrids, as well as to trace the origin of the allotetraploid *A. castaneoviride.* Hereafter, sterile hybrids of *A. incisum* and *A. ruprechtii* were named *A.* × *castaneoviride* with ploidy levels (e.g., 2x or 3x) to distinguish them from *A. castaneoviride* (allotetraploid hybrid species) in the present study.

## Materials and methods

### Sampling and DNA extraction

Four species (*A. incisum*, *A. ruprechtii*, *A. castaneoviride*, and *A. bimixtum*) and sterile hybrids were collected from 14 populations throughout Korea and transplanted into a greenhouse at Chungbuk National University (**Supplementary Table 1**). All voucher specimens were deposited in the herbarium of the Chungbuk National University (CBNU) and their accession numbers were described in **Supplementary Table 2**. Genomic DNAs were extracted from fresh leaves using a DNeasy Plant Mini Kit (Qiagen, Hilden, Germany) following the manufacturer’s protocol.

### Genome size estimation

After checking the ploidy levels of all plant materials, the genome sizes of *A. incisum, A. ruprechtii*, and their hybrid progenies (2x, 3x, and 4x) were measured using live materials selected from the sampled populations, which includes all hybrids (**Supplementary Figure 1**, **Supplementary Table 2**). 4x *Solanum tuberosum* with a 1c-value of 2.1 (Bennett and Smith, 1976) was used as the standard reference for calculating the genome size. Only 1 × 1 mm^2^ of the reference leaf and one-tenth leaf of *Asplenium* species were chopped together with 500 μl of nuclei extraction buffer using a CyStain UV Precise P kit (Sysmex Partec, Germany) in a Petri dish placed on ice. The debris was filtered using a non-sterile CellTrics filter Green 30 μm (Sysmex Partec), and nuclei stained with the staining buffer in CyStain UV Precise P kit (Sysmex Partec). The particle size was measured at least three times per sample using a CyFlow Cube 6 (Sysmex Partec).

### Plastome sequencing, assembly, and annotation

NGS libraries for four high-quality DNAs were constructed (Macrogen, Seoul, Korea) and sequenced using Illumina HISeq X Ten. The raw reads were trimmed by trimmomatic 0.36 (Bolger et al., 2014) using the following options: leading, 10; trailing, 10; sliding window, 4:20; and minlen, 50. The assembly method was identical to that of Kim et al. (2015) using *A. nidus* (NC_045119.1) and *A. prolongatum* (NC_035838) as reference sequences. Genes in plastome sequences were annotated and compared with the genes of *A. nidus* and *A. prolongatum* using Geneious 10.2.6 (Kearse et al., 2012) and confirmed using BLAST search (Altschul et al., 1990) and tRNAscan-SE (Lowe and Eddy, 1997).

### Amplification of *pgiC, rbcL,* and *rpoC2-rps2* regions

The gene encoding cytosolic phosphoglucose isomerase, *pgiC*, merits phylogenetic reconstruction in seed plants (Gottlieb and Ford, 1996) and is generally translated from a single copy gene, except in certain lineages (Gottlieb, 1982; Thomas et al., 1993). A codominant nuclear DNA marker using *pgiC* for homosporous ferns was developed to estimate mating and to study population genetics (Ishikawa et al., 2002) and has been applied to various fern lineage studies (Ebihara et al., 2005; de Groot et al., 2011; Dyer et al., 2012; Sessa et al., 2012).

We used a pair of primer sets for *pgiC* designed by Ishikawa et al. (2002) and *rbcL* designed by Schuettpelz and Pryer (2007) and de Groot et al. (2011) to verify the hybridization pattern between *A. incisum* and *A. ruprechtii*. Only individuals confirmed to have both types of *pgiC* were tested for the *rbcL.* To detect large deletions (2,139 bp) between *rpoC2* and *rps2* in *A. ruprechtii*-type plastomes, we designed a pair of primers (ARuRPS2: AGTGGATTCCTGCTGCCATC, ARuRPOC2: TGAGGGATTGAGTCGGCAAC) and applied them to all *A. bimixtum*, *A. ruprechtii*, and hybrids between *A. incisum* and *A. ruprechtii*.

The polymerase chain reaction (PCR) conditions for these loci were as follows: a 5-min denaturation step at 95°C, followed by 35 cycles at 95°C for 45 s, 50–53°C for 30–45 s, and 72°C for 60–75 s, followed by a 5-min final extension step at 72°C. The PCR products were purified using a PCR purification kit (Geneall, Seoul, Korea) and sequenced using an ABI 3730×l System (Macrogen).

### Phylogenetic analysis

A total of 68 *rbcL* sequences from *A. incisum* (17), *A. ruprechtii* (28), and their hybrids, including *A. bimixtum* (23), and 5 from *A. oligophlebium*, *A. boreale*, *A. normale*, *A. tripteropus*, and *A. trichomanes* were aligned using MAFFT (Katoh et al., 2002). All the *rbc*L sequence data generated in the present study were deposited in the NCBI database (**Supplementary Table 3**). Phylogenetic analysis was performed by the maximum likelihood method using IQ-TREE (Nguyen et al., 2015). The best-fit model for base substitution was selected by Bayesian information criterion using ModelFinder (Kalyaanamoorthy et al., 2017), and branch supports were accessed using UFBoot2 (Hoang et al., 2018).

### Identification of enlarged noncoding regions in the plastomes of *A. incisum* and *A. ruprechtii*

The *rpoC2-rps2* regions of the two plastomes, excluding from −1 to −100 upstream of *rps2*, were blasted against nucleotide collection using BLASTn (Altschul et al., 1990) with an e-value of 1e^−3^ and a word size of 11.

## Results

### Ploidy analyses of individuals based on the nuclear genotype and genome size measurement

**Figure 2:**
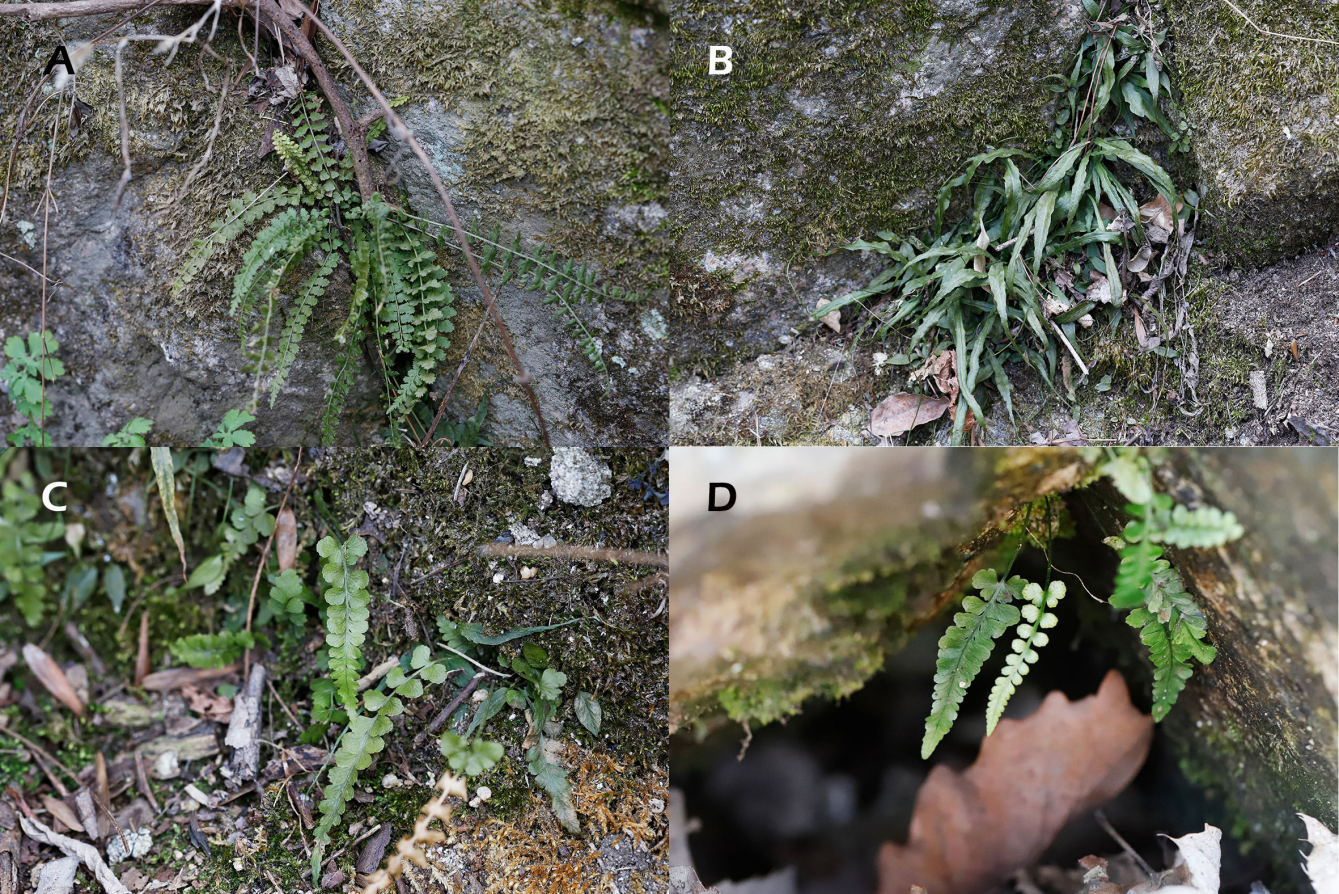
Plants and their natural habitats analyzed in the present study. **(A)** *A incisum*; **(B)** *A ruprechtii*; **(C)** *A castaneoviride*; **(D)** *A bimixtum*.

Based on leaf morphology, the plant samples were first separated into four groups (**Figure 2**, **Supplementary Table 1**): *A. incisum* (17 individuals), *A. ruprechtii* (28), hybrid (18), and *A. bimixtum* (5). All individuals morphologically identified as *A. incisum* and *A. ruprechtii* had homozygous nuclear genotypes. Meanwhile, sequencing traces of *pgiC* from hybrids showed a mixed template pattern with two peaks found at the same location. For an additional check of the correct sequence at these sites, seven PCR products (including an *A. bimixtum* individual) from 23 hybrids were cloned and confirmed to have both nuclear genotypes of *A. incisum* and *A. ruprechtii*.

**Figure 3:**
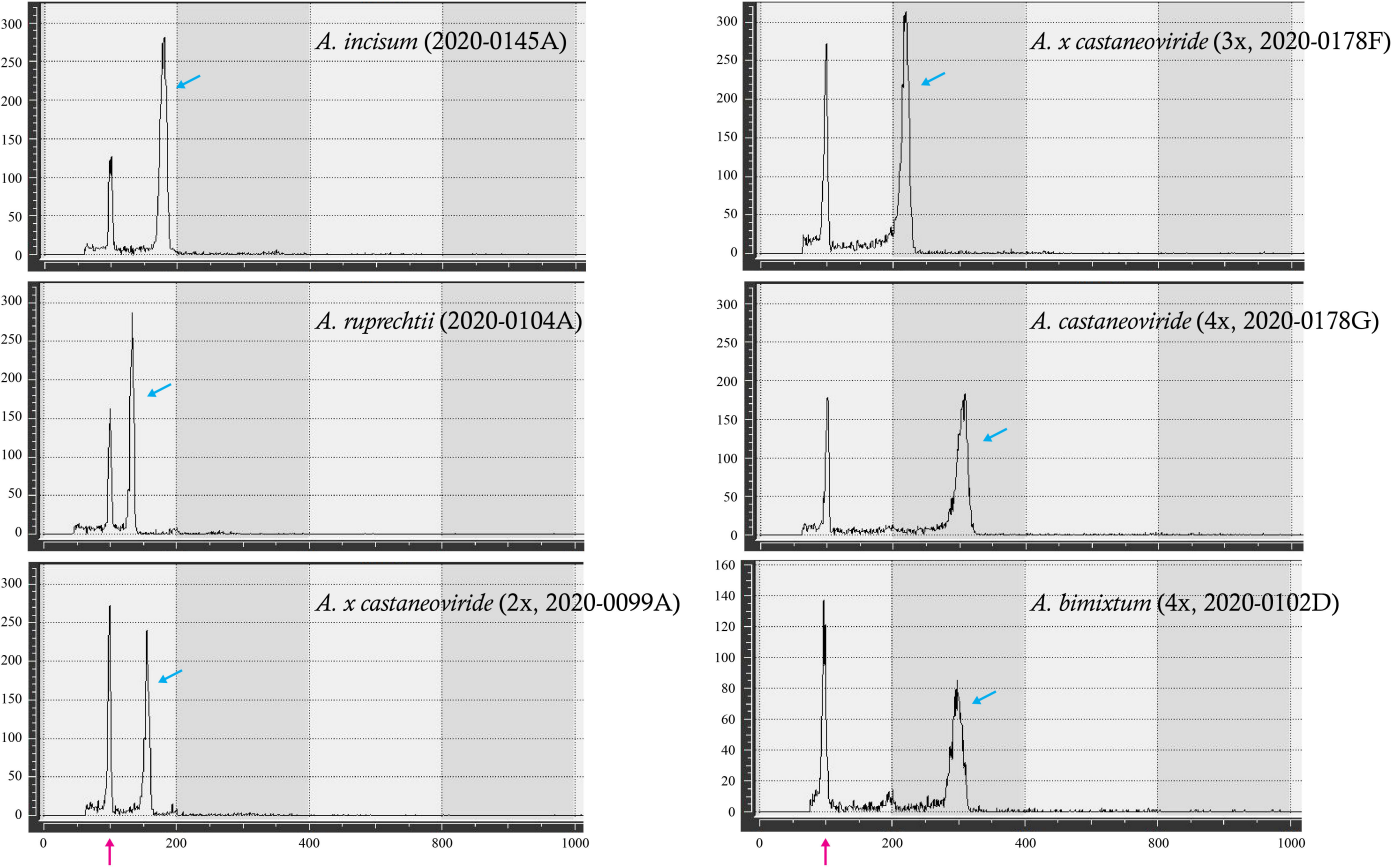
Examples of flow cytometry histograms of *A. incisum*, *A. ruprechtii*, and their hybrids. The *X-* and *Y*-axes show a relative fluorescence intensity and number of particles, respectively. Red and blue arrows refer to reference (*S. tuberosum*) and sample peaks, respectively.

Based on these three nuclear genotypes of *pgiC* (homozygous *A. incisum*, homozygous *A. ruprechtii*, and heterozygous genotypes of both) and the different genome sizes detected among the heterozygotes, they were grouped into six types. The average genome sizes of *A. incisum* and *A. ruprechtii* known as diploid were 3.75 pg and 2.79 pg, respectively, compared to the reference standard of *S. tuberosum*, (**Figure 3**, **Table 1**, **Supplementary Table 2**). Among the hybrids, the average genome size of 11 individuals in Buramsan and one in Bukhansan was 6.51 pg, which was close to the sum of the two parental species. These individuals were considered as allotetraploid *A. castaneoviride* because they had normal viable spores. In contrast, the average genome size of 10 hybrid individuals collected from five different populations was 3.28 pg, which is approximately close to the mean genome size of *A. incisum* and *A. ruprechtii*. These individuals were considered sterile diploid hybrid *A. × castaneoviride* (described as *A. × castaneoviride* 2x) because they had abortive spores. Only one triploid hybrid collected from Buramsan had a genome size of 4.62 pg, resembling the mean genome size of an allotetraploid *A. castaneoviride* and a diploid-origin *A. ruprechtii*. This triploid also had abortive spores, and was considered to be *A. × castaneoviride* 3x. The four individuals collected from Seongsan, known as *A. bimixtum*, had an average genome size of 6.32 pg with normal spores.

**Table 1:** Summary of genome size comparison for *A. incisum*, *A. ruprechtii*, and their hybrid progenies.

Taxon (No. of samples)	1 C-value (Mean ± SD)	Ploidy
*Asplenium incisum (4)*	3.756 ± 0.061094 (min. 3.668–max. 3.803)	2x
*Asplenium ruprechtii (5)*	2.793 ± 0.0307999 (min. 2.761–max. 2.835)	2x
*Asplenium* x *castaneoviride* 2x (10)	3.284 ± 0.0646841 (min. 3.204–max. 3.413)	2x
*Asplenium* x *castaneoviride* 3x (1)	4.624	3x
*Asplenium castaneoviride (12)*	6.512 ± 0.1706216 (min. 6.251–max. 6.734)	4x
*Asplenium bimixtum (4)*	6.323 ± 0.0921532 (min. 6.257–max. 6.435)	4x

### Genome structure and gene contents of plastomes in *Asplenium* species

The four plastomes of *Asplenium* species newly reported in the present study were from 150,899 (*A. × castaneoviride* 2x) to 153,116 bp (*A. incisum*) in length with a GC content of 40.8%–41.2% (**Table 2**). The length variation among the five plastomes including *A. bimixtum* was higher in the large single-copy (LSC) region than in the other regions because of the 2,139-bp deletion found in the plastome of *A.* × *castaneoviride* 2x. The LSC-IR boundaries were highly conserved, but the SSC-IR boundary of *A. incisum* was slightly different from that of the others because of the 22-bp IR expansion into *chlL* (**Supplementary Figure 2**).

**Table 2:** Summary of the plastomes of *Asplenium* species.

Species	Total length (bp)	LSC (bp)	SSC (bp)	IR (bp)	GC content (%)	Coverage depth (X)
*Asplenium incisum*	153,116	86,520	21,452	22,572	40.8	238.8
*Asplenium ruprechtii*	153,066	86,396	21,508	22,581	41.2	701.2
*Asplenium* x *castaneoviride* (2x)	150,899	84,260	21,517	22,561	41.2	448.8
*Asplenium castaneoviride*	153,071	86,400	21,509	22,581	41.0	348.8
*Asplenium* x *bimixtum* ^a^	153,098	86,394	21,508	22,598	41.2	294

a Downloaded from Ha et al. (2020).

*Asplenium castaneoviride*, *A. bimixtum*, and *A. × castaneoviride* (2x) have *A. ruprechtii*-type plastomes, whereas *A. incisum* always has its own plastome. Pairwise comparison of the whole plastome sequences showed that *A. ruprechtii*-type plastomes had high identities to each other with more than 98.48%, and increasing up to 99.88% when a large deletion that occurred in *A.* × *castaneoriviride* (2x) was excluded (**Supplementary Table 4**). In addition, two small inversions and a tandem repeat were found among *A. ruprechtii*-type plastomes (**Supplementary Figure 3**).

In contrast to the results of Lee et al. (2019), the plastome of *A. bimixtum* was clearly *A. ruprechtii*-type and not belonging to *A. incisum*-type. Therefore, the *rbcL* regions of four individuals of *A. bimixtum* were additionally sequenced and confirmed that they were identical to the *A. ruprechtii*-type *rbcL* without any exception.

### New finding of the enlarged noncoding region in the plastomes

The enlarged noncoding regions of *rpoC2-rps2* in the plastomes of *A. incisum* and *A. ruprechtii* and their hybrids were newly found compared to the previously reported plastomes in other ferns (Kim and Kim, 2020). Although 100 bp upstream of *rps2* was highly conserved within the genus, the intergenic spacer of *rpoC2-rps2* of *A. incisum*, *A. ruprechtii*, and their hybrids was 1,870–4,277 bp longer than that of other species with non-expanded plastomes (**Table 3**). Among the enlarged regions in *rpoC2-rps2* of *A. incisum, A. reprechtii*, and their hybrids, a large deletion of 2,139 bp was detected in the plastome of *A.* × *castaneoviride* 2x. We checked the presence/absence of this large deletion in all collected samples and confirmed that this event also occurred in other individuals from the Buramsan populations: five out of nine in *A. ruprechtii*, five out of six in *A.* × *castaneoviride* (2x), one in *A.* × *castaneoviride* (3x), and one out of five in *A. castaneoviride*.

**Table 3:** Enlarged noncoding regions (gray cells) in *Asplenium* plastomes. (*Bold accession numbers indicate the plastomes that were newly determined in the present study).

Taxon	Acc.	Enlarged noncoding regions							
		Region	Len (bp)	Region	Len (bp)	Region	Len (bp)	Region	Len (bp)
***Asplenium nidus***	NC_045119	*ycf12-trnG*	7,764	*rrn16-rps12*	1,219	*ycf2-trnN*	671	*rpoC2-rps2*	107
*Asplenium pekinense*	NC_035837		129		3,337		670		108
*Asplenium prolongatum*	NC_035838		238		1,227		1,969		108
*Asplenium incisum*	OP345470*		229		1,225		622		4,485
*Asplenium ruprechtii*	OP345473*		223		1,029		685		4,117
*Asplenium* x *castaneoviride*	OP345472*		223		1,029		685		1,978
*Asplenium castaneoviride*	OP345471*		223		1,029		685		4,122
*Asplenium* x *bimixtum*	MN912732		223		1,029		685		4,117

**Figure 4:**
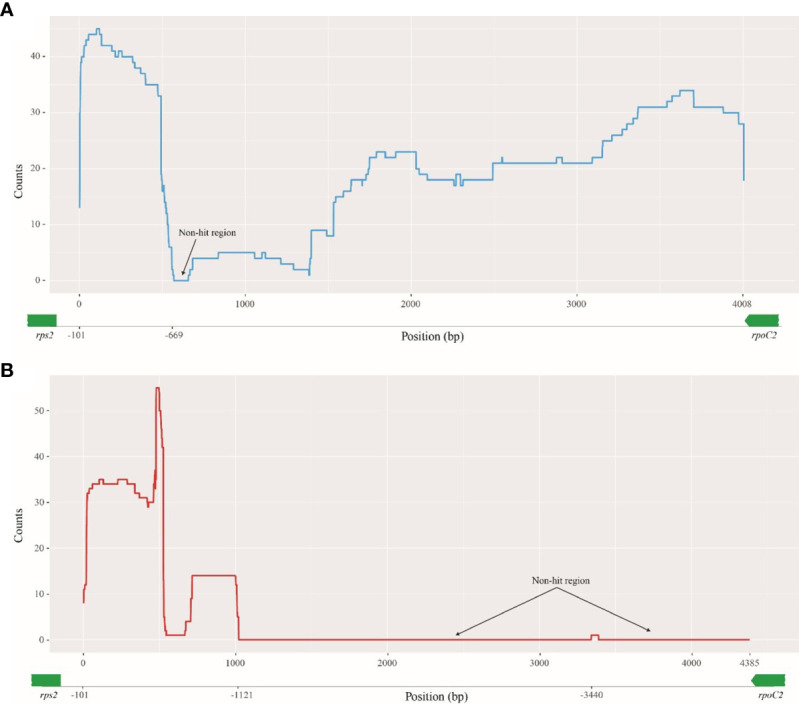
Blast results of enlarged regions against nucleotide collection. The *Y*-axis shows the number of hits to subject sequences and the *X*-axis shows the position of *rpoC2-rps2* regions. **(A)** *rpoC2-rps2* of *A. ruprechtii*; **(B)** *rpoC2-rps2* of *A. incisum*.

The enlarged DNAs of *A. incisum* and *A. ruprechtii* were originated from other sources even though they are very closely related taxa. The BLAST results for the *A. ruprechtii rpoC2-rps2* region (from −101 to −4,108 upstream of *rps2*) showed that most regions were hit at least once in 48 organisms tested consisting of 47 ferns and one fungi (**Figure 4A** and **Supplementary Table 5**); only the 87-bp region had no hits against the nucleotide collection. In contrast to the *A. ruprechtii rpoC2-rps2* region, the 1,021-bp region (from −101 to −1,121 bp upstream of *rps2*) of the *A. incisum rpoC2-rps2* region was only hit in the fern plastomes. Although the 49-bp region positioned at −3,440 bp upstream of *rps2* hit the mitochondrial genome of the fungus, this region was new in this plastome (**Figure 4B**).

### Maternal origin of hybrids derived from *A. ruprechtii* and *A. incisum*

To confirm the maternal origin of the hybrid progenies between *A. ruprechtii* and *A. incisum*, the *rbcL* sequences of 68 collected samples were compared. All conspecific *rbcL* sequences of *A. ruprechtii* (28 individuals) and *A. incisum* (17 individuals) were almost identical and clearly divided, even though one individual of *A. incisum* had 1-bp substitution against others. All hybrids, *A.* × *castaneoviride* (2x), *A.* × *castaneoviride* (3x), *A. castaneoviride*, and *A. bimixtum*, had an *A. ruprechtii*-type *rbcL*, except for two individuals of *A.* × *castaneoviride* (2x), which shared 1-bp substitution against the other *A. ruprechtii* individuals. In the phylogenetic analysis, they were located in the same clade with their maternal origin *A. ruprechtii* (**Supplementary Figure 4**).

## Discussion

### Hybridization pattern between *A. ruprechtii* and *A. incisum*

*Asplenium* L. tends to hybridize and polyploidize (Dyer et al., 2012; Chang et al., 2018; Liang et al., 2021) in natural populations. Recent experiments based on maternally inherited plastid genes showed that some allotetraploids of the genus (e.g., *A. billotii* and *A. foreziense*) have reciprocal parentage. However, other species such as *A. adiantum-nigrum* and *A. balearicum* have no evidence of reciprocal origins in the *obovatum-adiantum-nigrum* group because the former two tetraploids were embedded together in the same clade with respective diploid parents, whereas the latter two tetraploids formed a clade with only maternal diploid of *A. onopteris* (Sessa et al., 2018). It is possible to assume the path of hybridization using the positions of hybrids and their parental species in the phylogenetic tree, to get insights into the evolution of species over the past, present, and future. For instance, in New Zealand, two octoploids, *A. cimmeriorum* and *A. gracillimum*, are reported to result from reciprocal hybridization between two tetraploids, *A. bulbiferum* and *A. hookerianum* (Shepherd et al., 2008).

Considering that *A.* × *castaneoviride* (2x or 3x) and *A. ruprechtii* grow side-by-side in Korea, *A. ruprechtii* is expected to be a maternal parent of *A.* × *castaneoviride*. At this point, we have shelved the possibility of allotetraploid formation *via* a triploid bridge because only one individual of triploid *A.* × *castaneoviride* was observed in nature with abortive spores. Our results showed that all hybrids have two types of *pgiC* originated from *A. ruprechtii* and *A. incisum*, but they have only *A. ruprechtii*-type plastome genes of *rbcL*. The nuclear single-copy and plastome genes showed completely unidirectional hybrid formation between *A. ruprechtii* and *A. incisum* (χ^2 = ^7, df = 1, *p* < 0.01). Therefore, *A. castaneoviride*, a consequence of polyploidization by diploid hybrid *A.* × *castaneoviride*, is also expected to have *A. ruprechtii*-type plastome sequence affecting hybridization between *A. ruprechtii* and *A. incisum*.

Gastony and Yatskievych (1992) showed that sexual species play the role of female parent when hybridization occurs between an apogamous and a sexual species in ferns because functional archegonia is lacking in an apogamous species. However, both *A. ruprechtii* and *A. incisum* are known to be sexual species, and the sporangium in both species really exhibited 64 spores in our observations. Vogel et al. (1998b) inferred that the predominantly unidirectional fertilization between *Asplenium trichomanes* and *A. septentrionale* is related to breeding systems. According to them, the obligately inbreeding taxon may have difficulty playing the role of a maternal parent in the hybridization process because of the synchronous maturity of male and female gametes. Based on the length variation due to the large deletion of *rpoC2-rps2* in Buramsan populations of *A. ruprechtii* and tetraploid *A. castaneoviride* and their capacity to hybridize with other species in the same genus (Lee et al., 2019), it is suggested that *A. ruprechtii* is an outbreeding taxon. In contrast, *A. incisum* has hybridized with species other than *A. ruprechtii* and acts as a maternal parent (Ebihara, 2016). Therefore, breeding systems might not play an important role in the female parent of *A. ruprechtii*. If the ontogenetic sequence of gametangia differs between the two parental species, one species beginning with male prothalli may be able to reach the male parent of another species beginning with female prothalli, such as dichogamy in angiosperms. However, gametophytes of *A. ruprechtii* and *A. incisum* first produce archegonia, followed by antheridia (Jeong and Lee, 2006).

As mentioned above, *A. ruprechtii* and *A. incisum* have the same breeding system of outcrossing, sexual reproductive mode, and diploid level. Nevertheless, it is not known why only *A. ruprechtii* plays the role of a female parent in this asymmetric hybridization.

One possible scenario for this asymmetric hybridization may be the sex-determination system in ferns. Antheridiogens are pheromones secreted by mature female fern gametophytes, which induce neighboring asexual gametophytes to produce precocious antheridia (Schneller and Ranker, 2008). Hornych et al. (2021) showed that all four species tested in *Asplenium* responded to conspecific antheridiogen sources, and several species in various lineages responded to these sources. Therefore, it is likely that antheridiogens in genus *Asplenium* are important for sex determination. If *A. incisum* gametophytes can inhabit the *A. ruprechtii* population, but not vice versa, antheridiogens of *A. ruprechtii* can induce *A. incisum* gametophytes to grow male gametophytes owing to environmental conditions such as light and moisture. Then, the mature sperm of *A. incisum* may arrive at the archaegonia of *A. ruprechtii* to produce a hybrid. Since the available data are insufficient for this scenario, further investigation of asymmetric hybridization between *A. ruprechtii* and *A. incisum* is required, including a glasshouse experiment under restricted conditions, because both species have frequently hybridized.

### Multiple origins of allotetraploid *A. castaneoviride*

*Asplenium castaneoviride*, which is distributed in China, Japan, and Korea (Lin and Viane, 2013) is an allotetraploid species derived from *A. ruprechtii* and *A. incisum*. However, the origin of this allotetraploid species has not been discussed.

**Figure 5:**
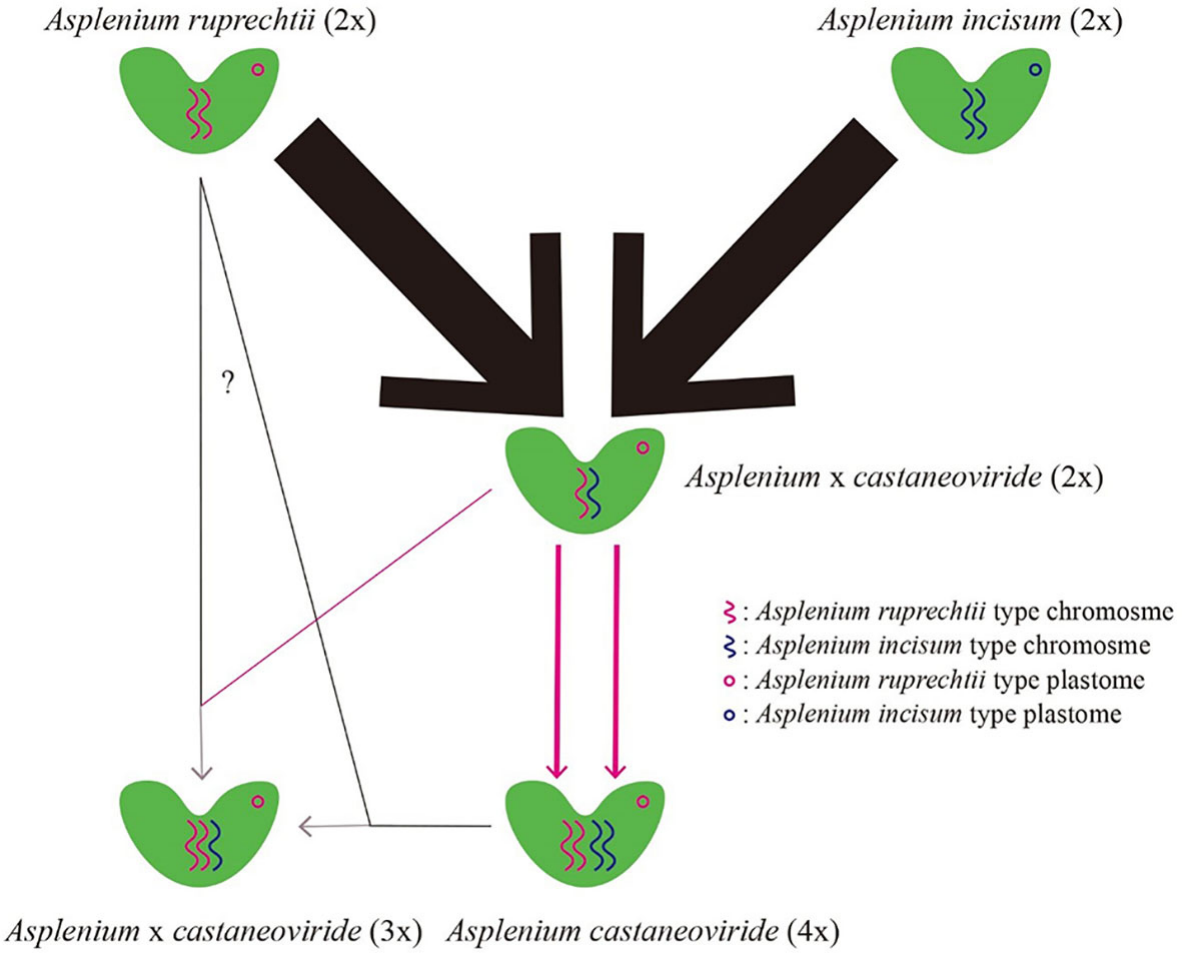
Hypothetical origins of different ploidy levels of diploid, triploid, and tetraploid *Asplenium castaneoviride.* Red and black lines refer to unreduced diplospory and reduced haplospory, respectively. Line thickness represents the frequency of hybrid individuals found in this study.

Multiple allopolyploidization events, in which progenitor species are repeatedly hybridized and independently polyploidized, can produce intraspecific variations within its progenitor species (Sigel, 2016). A large deletion of 2,139 bp that occurred in *rpoC2-rps2* was not found in any *A. ruprechtii* individuals, except for the Buramsan populations (P4) where this mutation may be caused independently. We proposed a hypothesis to induce the allotetraploid *A. castaneoviride* from the diploid hybrid between *A. ruprechtii* and *A. incisum* (**Figure 5**). A haploid gamete of *A. ruprechtii* may form a zygote with heterozygous diploid gametes of diploid *A.* × *castaneoviride* or *A. castaneoviride*, and a large deletion of *rpoC2-rps2* remains at different ploidy levels (2x, 3x, and 4x) of *A. castaneoviride* in Buramsan populations. Consequently, the allotetraploid *A. castaneoviride* was independently produced by more than two maternal origins in the Buramsan population.

Although the allotetraploid *A. castaneoviride* was fertile, we did not find a population consisting of only *A. castaneoviride* without the maternal parent of *A. ruprechtii* in Korea.

Allopolyploids generally have advantages such as heterosis, an adaptive advantage of progeny (Birchler et al., 2010), and gene redundancy masking recessive alleles (Comai, 2005) compared to their progenitors. However, minor cytotypes can be excluded from the population by forming sterile odd-ploidy offspring (Levin, 1975). In Korea, *A. ruprechtii* and *A. incisum* are widely distributed, and hybridization frequently occurs between them. During the study, we found four diploid hybrid populations with sympatric distribution of both parental species *A. ruprechtii* and *A. incisum*, but allotetraploid species were found in only two populations of Bukhansan (P2) and Buramsan (P4). Although we did not include the plant samples from other ranges of the species like the Chinese one in the present study, we propose that the driving force for the polyploidization of diploid hybrids (*A.* × *castaneoviride*) may have been maintained, but restricted to the natural population by a frequency-dependent mating disadvantage (Husband, 2000).

### Is *Asplenium* × *bimixtum* a hybrid among the three species?

*Asplenium* x *bimixtum* C. S. Lee & K. Lee ex Y.H. Ha was first reported by Lee et al. (2015) based on its morphological characteristics and is endemic to Korea. Lee et al. (2015) argued that this species was formed by multiple hybridizations among three species: *A. ruprechtii*, *A. trichmanes*, and *A. incisum*. They attempted to prove the occurrence of these multiple hybridizations based on 1C-value measurements and molecular phylogenetic analyses (Lee et al., 2019). However, they ignored that the standard plant should run together along with running samples to standardize genome size and cover the instability of the equipment. This may explain why the calculated genome sizes of certain species showed a high standard deviation in their study. In addition, they considered all *A. castaneotiride* individuals to be allotetraploids, but we confirmed the presence of triploid *A.* × *castaneoviride* and tetraploid *A. castaneoviride* from the comparison of 1C-values. Furthermore, the 1C-values of *A*. *bimixtum* ranged within those of the allotetraploid *A. castaneoviride* in their results as well as in this study. Lee et al. (2019) also argued that *A. bimixtum* has two types of plastid genes: *A. ruprechtii*-type *rbcL* and *A. incisum*-type *rps4*. However, the complete plastome sequence of *A. bimixtum* is more similar to *A. ruprechtii* plastome with 99.96% identity (**Supplementary Table 4**) and has the same type of *rps4-trnS*. The four individuals of *A. bimixtum* used in the present study were all embedded in the *A. ruprechtii* clade in the phylogenetic tree (**Supplementary Figure 4**). Although we cannot completely rule out the possibility of overlooking *A*. x *bimixtum* in the habitat, this species may be included in the variation of *A. castaneoviride*, and it will be more apt to treat it as a synonym of allotetraploid *A. castaneoviride* with the available data. If we recognize *A*. *bimixtum* as a member of the alltotetraploid of *A. castaneoviride*, it could be concluded that *A. castaneoviride* has derived from more than three origins based on their genetic variation patterns: (1) normal type without large deletion in *rpoC2-rps2*, (2) with large deletion in *rpoC2-rps2*, and (3) with a small inversion of *trnN-ndhF* and more tandem repeats in *ycf2* (*A. bimixtum* type) in Korea, even though they are grouped together in the same clade as their maternal origin.

### Enlarged noncoding region in fern organelles found in *Asplenium* species

Robison et al. (2018) suggested the concept of mobile open reading frames in fern organelles (MORFFO), because several ORFs varying in sequence length, insertion position, and gene arrangement are widely distributed in Pteridaceae plastomes. In contrast, the earliest diverging group with these MORFFOs is in Ophioglossaceae, and six polycistronic genes in the plastome of *Mankyua chejuense* appear to be the original form of non plastome foreign DNAs in fern plastomes (Kim and Kim, 2018).

Certain flanking regions of MORFFOs are highly divergent or unique though these appear to be conserved throughout the fern lineage if they exist. For instance, the *rps12-rrn16* regions of *Odontosoria chinensis* plastome containing *morffo* had 94.3% of query coverage hit to nucleotide collection (Robison et al., 2018); however, the *trnE-psbM* region, which was also expanded by foreign DNAs in the same plastome, had only 45% of query coverage. Similar cases are reported in other fern genera. The *trnV-rrn16* regions of *Hymenophyllum* plastomes were expanded because of foreign DNA insertions (Kim and Kim, 2020); however, only 33.6% of foreign DNAs of *H. polyanthos* were hit by *H. coreanum* using BLAST, although these two species are closely related to each other.

In the present study, we also found foreign DNA in the *rpoC2-rps2* region of the *A. incisum* plastome, which has not been reported so far, and confirmed that it originated from a completely different source. To recognize and describe this feature on the plastome, similar to the *rpoC2-rps2* of *A. incisum*, in which no *morffo* is observed, we propose the term of “Enlarged Noncoding Region in Fern Organelles (ENRIFO),” because many enlarged noncoding regions in fern organelles include unique sequences.

To understand the origin of ENRIFO in *A. incisum*, one should understand how it is completely different from *A. ruprechtii*. Kim and Kim (2020) suggested that palindromic sequences, including tRNA genes, are strongly related to the genomic instability of fern plastomes, and long foreign DNA is degraded under selective pressure. As the ENRIFOs of *Asplenium*, except for *A. incisum*, vary in position in the plastome and share certain sequences, it is possible that a common ENRIFO has changed its position in the plastomes of the genus *Asplenium*. However, this cannot explain why unidentified sequences are found in the ENRIFOs within the *Asplenium* plastomes. The simplest explanation for these unidentified ENRIFOs is that these can be easily substituted by foreign DNA, which targets certain regions in the plastome associated with genomic instability. The positions of ENRIFOs generally vary in fern plastomes; however, they are found at the same position in closely related taxa (Kim and Kim, 2020). Considering the low similarity between ENRIFOs in closely related taxa, DNA recombination may have occurred more frequently than expected. To completely understand this genomic feature, further study of the genus *Asplenium* is needed.

## Data availability statement

The datasets presented in this study can be found in online repositories. The names of the repository/repositories and accession number(s) can be found below: https://www.ncbi.nlm.nih.gov/nuccore/OP345470.1/, https://www.ncbi.nlm.nih.gov/nuccore/OP345473, https://www.ncbi.nlm.nih.gov/nuccore/OP345472, https://www.ncbi.nlm.nih.gov/nuccore/OP345471.

## Author contributions

Conceptualization, HK and JK. Methodology, HK and SP. Sequencing, SP. Software and data curation, HK. Resources, SP, HK and JK. Writing—original draft preparation, HK. Writing—review and editing, HK and JK. Visualization, HK. Supervision, JK. Project administration, HK and funding acquisition, HK and JK. All authors contributed to the article and approved the submitted version.

## Supplementary material

Supplementary Figure 1 Map of the location of populations where the plant samples were collected and used to measure the genome size of *A. incisum, A. ruprechtii*, and their hybrid taxa. The population numbers are identical to the **Supplementary Table 1** and the red circles indicate the sympatric populations where the two parental species and their hybrid progenies occupied together.

Supplementary Figure 2 Comparison the border regions of the LSC, SSC, and IR among five plastomes.

Supplementary Figure 3 Small inversions and tandem repeats among *A. ruprechtii* type plastomes. A) Two small inversions found *trnN-ndhF* and *rps15-ycf1*, respectively. B) A variable number tandem repeat in *ycf2*.

Supplementary Figure 4 Phylogeny of *Asplenium incisum*, *A. ruprechtii* and their hybrids constructed by ML method using IQTREE. Numbers on the branch refer to ultrafast bootstrap support value. Number on the base substitution left indicates the position of base substitution on the alignment of *rbcL* (1283 bp in length).
