# Supplementary figures and images for: Dynamic hybridization between two spleenworts, Asplenium incisum and Asplenium ruprechtii in Korea
Source: Front Plant Sci. 2023 Jul 5;14:1116040. doi: 10.3389/fpls.2023.1116040 (PMC10354290; doi:10.3389/fpls.2023.1116040)

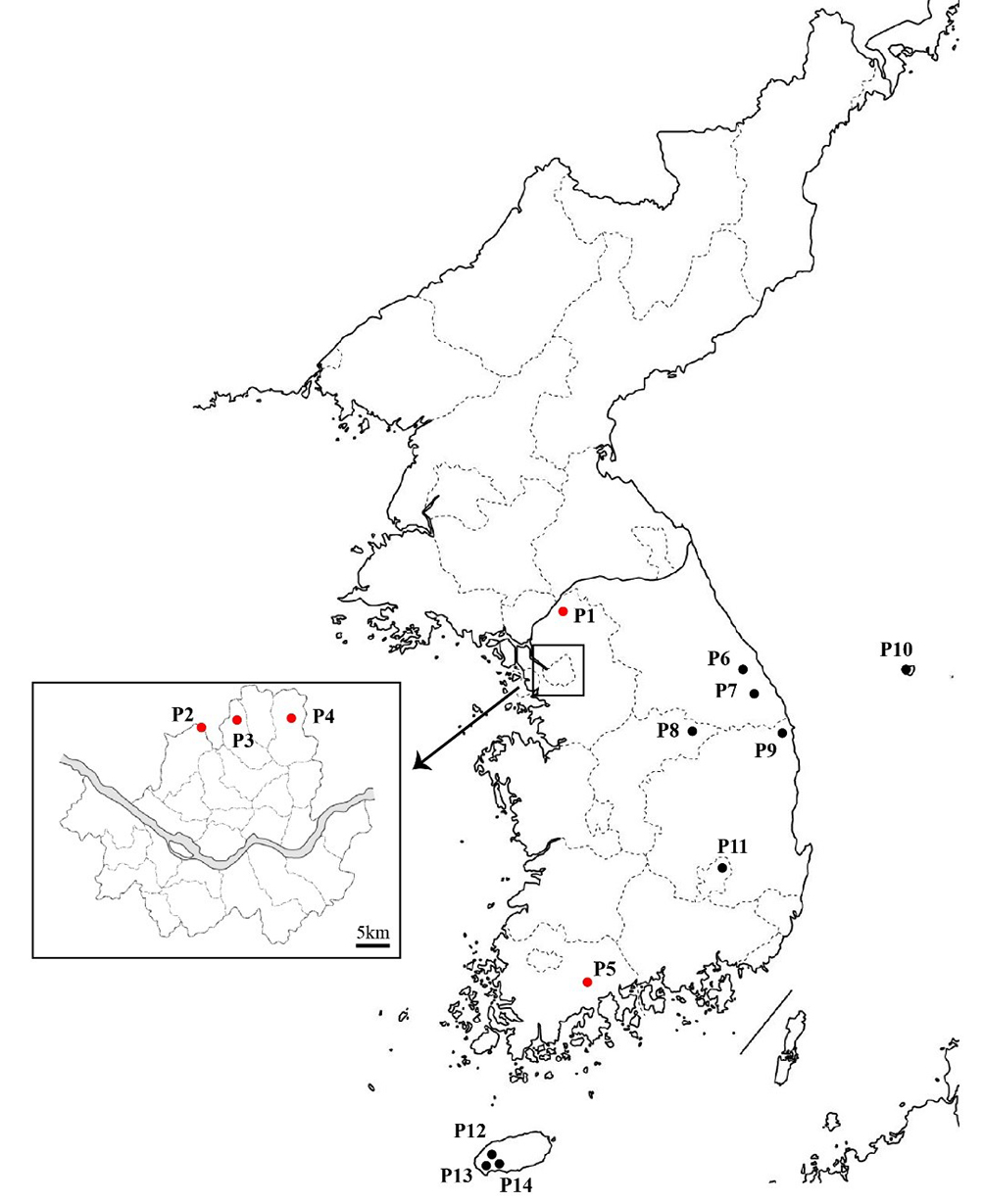

Supplement: Supplementary Figure 1 — Map of the location of populations where the plant samples were collected and used to measure the genome size of A. incisum, A. ruprechtii, and their hybrid taxa. The population numbers are identical to the Supplementary Table 1 and the red circles indicate the sympatric populations where the two parental species and their hybrid progenies occupied together. [file Image_1.jpeg]

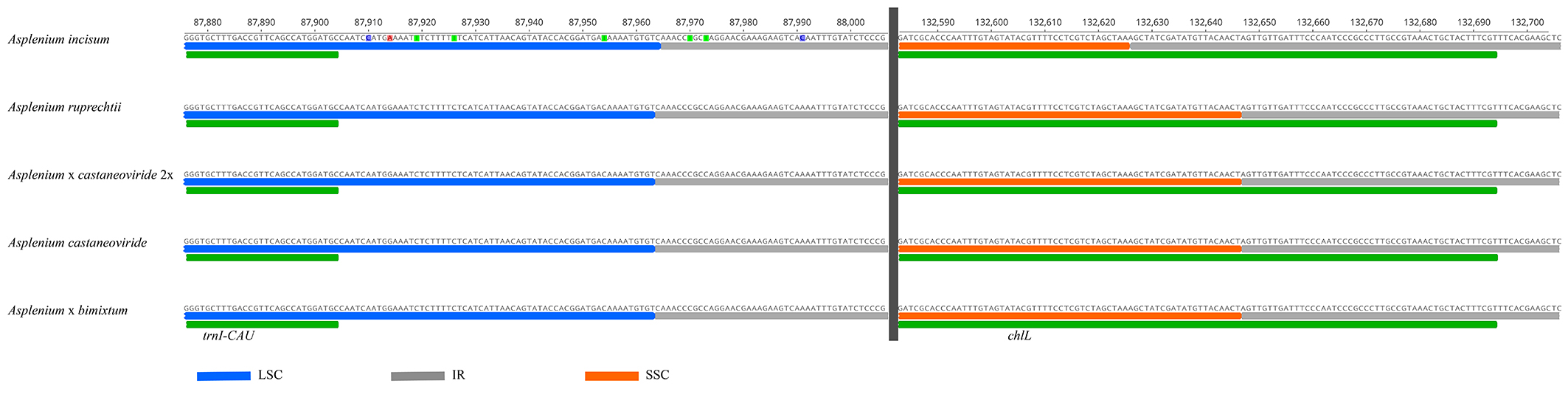

Supplement: Supplementary Figure 2 — Comparison the border regions of the LSC, SSC, and IR among five plastomes. [file Image_2.jpeg]

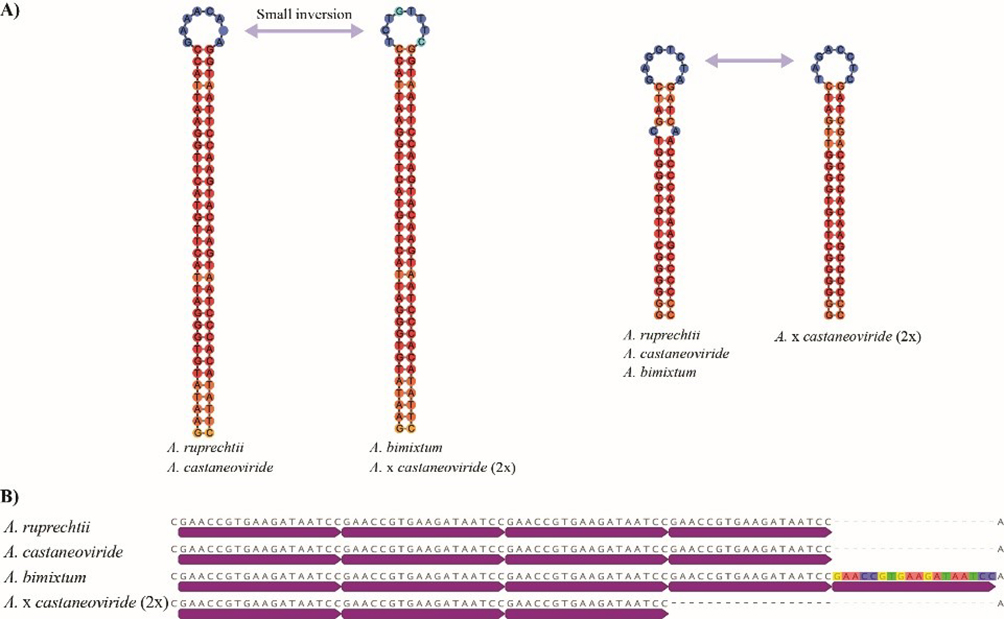

Supplement: Supplementary Figure 3 — Small inversions and tandem repeats among A. ruprechtii type plastomes. A) Two small inversions found trnN-ndhF and rps15-ycf1, respectively. B) A variable number tandem repeat in ycf2. [file Image_3.jpeg]

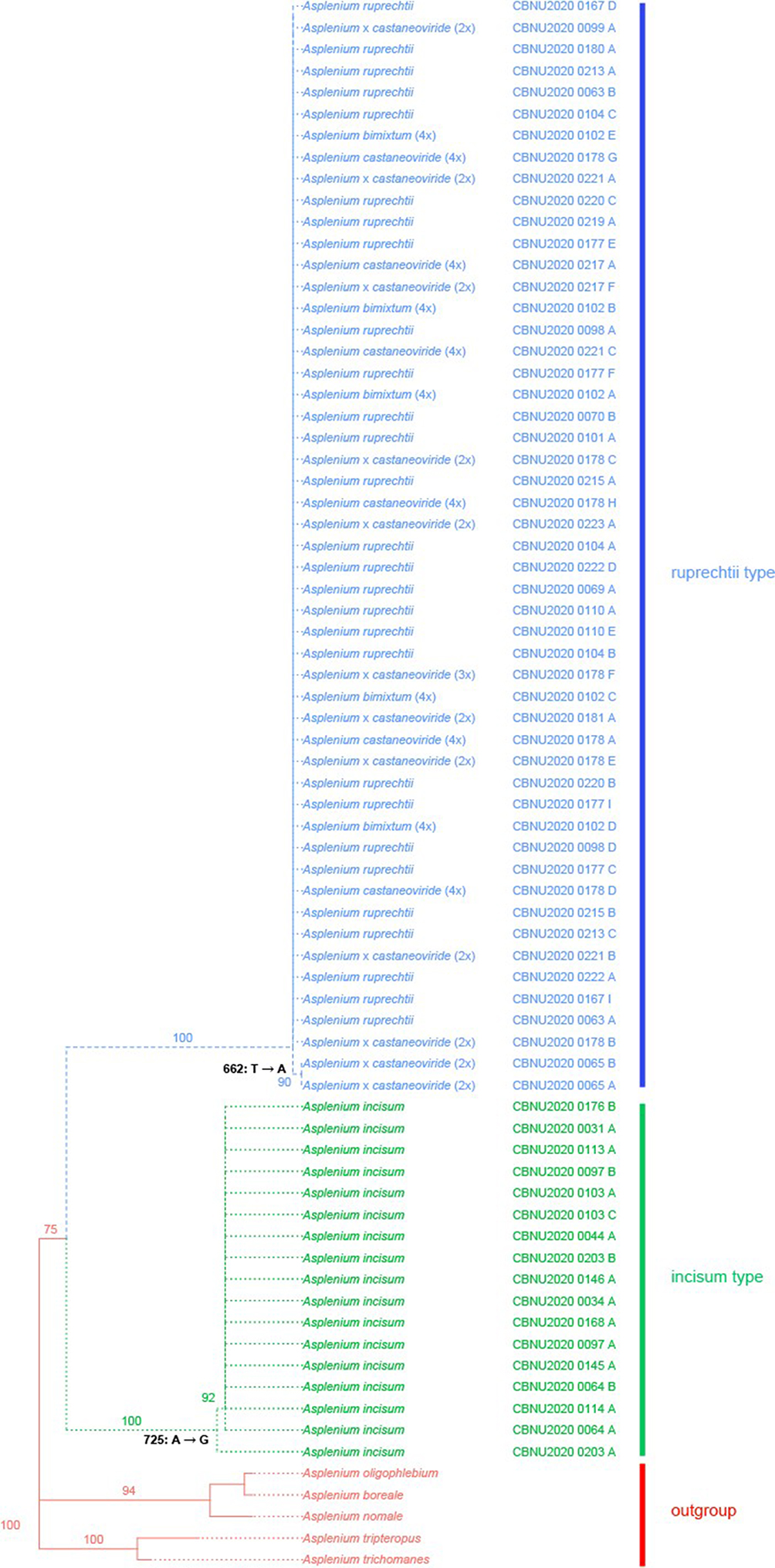

Supplement: Supplementary Figure 4 — Phylogeny of Asplenium incisum, A. ruprechtii and their hybrids constructed by ML method using IQTREE. Numbers on the branch refer to ultrafast bootstrap support value. Number on the base substitution left indicates the position of base substitution on the alignment of rbcL (1283 bp in length). [file Image_4.jpeg]
