# Supplementary material for: Dynamic hybridization between two spleenworts, Asplenium incisum and Asplenium ruprechtii in Korea
Source: Front Plant Sci. 2023 Jul 5;14:1116040. doi: 10.3389/fpls.2023.1116040 (PMC10354290; doi:10.3389/fpls.2023.1116040)
Supplement: Supplementary file 5 [file Table_1.docx]

| **Taxon** | **Collection site***  **(site number on Supplementary Fig. 4)** | **No. of individuals** | **Ploidy** |
| --- | --- | --- | --- |
| *Asplenium incisum* | **Seongsan, Gyeonggido (P1)** | 2 | 2x |
|  | **Uicheon, Seoul (P3)** | 2 |  |
|  | **Buramsan, Seoul (P4)** | 1 |  |
|  | Uljin, Gyeongsangbukdo (P9) | 2 |  |
|  | Ulleung, Gyeongsangbukdo (P10) | 2 |  |
|  | Dodong, Daegu (P11) | 1 |  |
|  | **Songgwangsa, Jeonlanamdo (P5)** | 2 |  |
|  | **Dololeum, Jeju (P12)** | 2 |  |
|  | Hwasun, Jeju (P14) | 2 |  |
|  | Sanyang, Jeju (P13) | 1 |  |
| *Asplenium ruprechtii* | **Seongsan, Gyeonggido (P1)** | 4 | 2x |
|  | **Bukhansan, Seoul (P2)** | 3 |  |
|  | **Uicheon, Seoul (P3)** | 2 |  |
|  | **Buramsan, Seoul (P4)** | 9 |  |
|  | Seokbyeongsan, Gangwondo (P6) | 2 |  |
|  | Deokhangsan, Gangwondo (P7) | 2 |  |
|  | Danyang, Chungcheongbukdo (P8) | 2 |  |
|  | Dodong, Daegu (P11) | 2 |  |
|  | **Songgwangsa, Jeonlanamdo (P5)** | 2 |  |
| *Asplenium* x *castaneoviride* | **Bukhansan, Seoul (P2)** | 4 | 2x |
|  | **Uicheon, Seoul (P3)** | 2 |  |
|  | **Buramsan, Seoul (P4)** | 4 |  |
|  | **Songgwangsa, Jeonlanamdo (P5)** | 1 |  |
|  | **Buramsan, Seoul (P4)** | 1 | 3x |
| *Asplenium castaneoviride* | **Bukhansan, Seoul (P2)** | 1 | 4x |
|  | **Buramsan, Seoul (P4)** | 5 |  |
| *Asplenium* x *bimixtum* | **Seongsan, Gyeonggido (P1)** | 5 | 4x |

**Supplementary Table 1.** The collection information of plant materials used for the present study. (*The boldic population were used for genome size comparison)
