# Supplementary material for: Dynamic hybridization between two spleenworts, Asplenium incisum and Asplenium ruprechtii in Korea
Source: Front Plant Sci. 2023 Jul 5;14:1116040. doi: 10.3389/fpls.2023.1116040 (PMC10354290; doi:10.3389/fpls.2023.1116040)
Supplement: Supplementary file 6 [file Table_2.docx]

**Supplementary Table 2.** Ploidy levels of *A. incisum*, *A. ruprechtii*, and their hybrid progeny.

| Taxon | Collection site* | Voucher number | Peak | | Ratio (S/R) | 1 C-value of subject | | | Ploidy level |
| --- | --- | --- | --- | --- | --- | --- | --- | --- | --- |
|  |  |  | **Reference** | Subject |  | 1C-value | Average | S.D. |  |
| *Asplenium incisum* | Songgwangsa, Jeonlanamdo (P5) | CBNU2020-0097 | 6682 | 11671 | 1.747 | 3.668 | 3.756 | 0.061094 | 2x |
|  | Seongsan, Gyeonggido (P1) | CBNU2020-0103 | 6482 | 11700 | 1.805 | 3.790 |  |  | 2x |
|  | Dololeum, Jeju (P12) | CBNU2020-0145 | 6439 | 11530 | 1.791 | 3.760 |  |  | 2x |
|  |  | CBNU2020-0146 | 6304 | 11417 | 1.811 | 3.803 |  |  | 2x |
| *Asplenium ruprechtii* | Songgwangsa, Jeonlanamdo (P5) | CBNU2020-0098 | 6207 | 8189 | 1.319 | 2.771 | 2.793 | 0.0307999 | 2x |
|  | Seongsan, Gyeonggido (P1) | CBNU2020-0101 | 6427 | 8449 | 1.315 | 2.761 |  |  | 2x |
|  |  | CBNU2020-0104 | 6468 | 8576 | 1.326 | 2.784 |  |  | 2x |
|  | Buramsan, Seoul (P4) | CBNU2020-0177A | 6541 | 8765 | 1.340 | 2.814 |  |  | 2x |
|  |  | CBNU2020-0177E | 6452 | 8709 | 1.350 | 2.835 |  |  | 2x |
| *Asplenium* x *castaneoviride* (2x) | Bukhansan, Seoul (P2) | CBNU2020-0181 | 6389 | 9814 | 1.536 | 3.226 | 3.284 | 0.0646841 | 2x |
|  | Uicheon, Seoul (P3) | CBNU2020-0065A | 6320 | 9849 | 1.558 | 3.273 |  |  | 2x |
|  |  | CBNU2020-0065B | 6467 | 10042 | 1.553 | 3.261 |  |  | 2x |
|  | Buramsan, Seoul (P4) | CBNU2020-0178B | 6598 | 10066 | 1.526 | 3.204 |  |  | 2x |
|  |  | CBNU2020-0178C | 6539 | 10162 | 1.554 | 3.264 |  |  | 2x |
|  |  | CBNU2020-0178E | 6386 | 9821 | 1.538 | 3.230 |  |  | 2x |
|  |  | CBNU2020-0217 | 6466 | 10508 | 1.625 | 3.413 |  |  | 2x |
|  | Songgwangsa, Jeonlanamdo (P5) | CBNU2020-0099 | 6407 | 10017 | 1.563 | 3.283 |  |  | 2x |
|  | Seongsan, Gyeonggido (P1) | CBNU2020-0221A | 6480 | 10356 | 1.598 | 3.356 |  |  | 2x |
|  |  | CBNU2020-0221A | 6446 | 10218 | 1.585 | 3.329 |  |  | 2x |
| *Asplenium* x *castaneoviride* (3x) | Buramsan, Seoul (P4) | CBNU2020-0178F | 6365 | 14014 | 2.202 | 4.624 | 4.624 | 0 | 3x |
| *Asplenium castaneoviride* | Buramsan, Seoul (P4) | CBNU2020-0178A | 6415 | 19404 | 3.025 | 6.352 | 6.512 | 0.1706216 | 4x |
|  |  | CBNU2020-0178D | 6498 | 19361 | 2.980 | 6.257 |  |  | 4x |
|  |  | CBNU2020-0178G | 6503 | 19525 | 3.002 | 6.305 |  |  | 4x |
|  |  | CBNU2020-0178H | 6558 | 19522 | 2.977 | 6.251 |  |  | 4x |
|  |  | CBNU2020-0217A | 6542 | 20979 | 3.207 | 6.734 |  |  | 4x |
|  |  | CBNU2020-0217B | 6415 | 20211 | 3.151 | 6.616 |  |  | 4x |
|  |  | CBNU2020-0217C | 6396 | 20113 | 3.145 | 6.604 |  |  | 4x |
|  |  | CBNU2020-0217D | 6362 | 20192 | 3.174 | 6.665 |  |  | 4x |
|  |  | CBNU2020-0217E | 6460 | 20178 | 3.124 | 6.559 |  |  | 4x |
|  |  | CBNU2020-0217G | 6496 | 20387 | 3.138 | 6.591 |  |  | 4x |
|  |  | CBNU2020-0217H | 6472 | 20256 | 3.130 | 6.573 |  |  | 4x |
|  | Bukhansan, Seoul (P2) | CBNU2020-0221 | 6411 | 20250 | 3.159 | 6.633 |  |  | 4x |
| *Asplenium bimixtum* | Seongsan, Gyeonggido (P1) | CBNU2020-0102A | 6411 | 19101 | 2.979 | 6.257 | 6.323 | 0.0921532 | 4x |
|  |  | CBNU2020-0102B | 6452 | 19166 | 2.971 | 6.238 |  |  | 4x |
|  |  | CBNU2020-0102C | 6099 | 18688 | 3.064 | 6.435 |  |  | 4x |
|  |  | CBNU2020-0102D | 6318 | 19137 | 3.029 | 6.361 |  |  | 4x |

*The parentheses after collection site is indicated the population number which were mapped on the Supplementary Figure 4.
