# Supplementary material for: Dynamic hybridization between two spleenworts, Asplenium incisum and Asplenium ruprechtii in Korea
Source: Front Plant Sci. 2023 Jul 5;14:1116040. doi: 10.3389/fpls.2023.1116040 (PMC10354290; doi:10.3389/fpls.2023.1116040)
Supplement: Supplementary file 7 [file Table_3.docx]

**Supplementary Table 3.** The accession numbers of the *Asplenium* *rbcL* gene sequences generated and used for the phylogenetic analysis in this study.

| Taxon | Voucher No. | Acc. No. |
| --- | --- | --- |
| *A. incisum* | CBNU2020 0031A ~ 0203B | OP329999~330014 |
| *A. ruprechtii* | CBNU2020 0063A ~ 0222D | OP330015~330041 |
| *A.* x *castaneoviride* (2X) | CBNU2020 0065A ~ 0223A | OP330042~330051 |
| *A.* x *castaneoviride* (3X) | CBNU2020 0178F | OP330052 |
| *A. castaneoviride* (4X) | CBNU2020 0102A ~ 0221C | OP330053~330062 |
| *A.* x *bimixtum* |  | MN912732 |
| *A. oligophlebium* | CBNU2020 0084A | OP330063 |
| *A. boreale* | CBNU2020 0075A | OP330064 |
| *A. normale* | CBNU2020 0206A | OP330065 |
| *A. tripteropus* | CBNU2020 0196A | OP330066 |
| *A. trichomanes* | CBNU2020 0100A | OP330067 |
