# Supplementary material for: Dynamic hybridization between two spleenworts, Asplenium incisum and Asplenium ruprechtii in Korea
Source: Front Plant Sci. 2023 Jul 5;14:1116040. doi: 10.3389/fpls.2023.1116040 (PMC10354290; doi:10.3389/fpls.2023.1116040)
Supplement: Supplementary file 8 [file Table_4.docx]

**Supplementary Table 4.** Percentages of identical bases among plastomes. The numbers in parentheses are percentages excluding the large deletions (2,139 bp) found in *Asplenium × castaneoviride* (2x).

|  | *Asplenium incisum* | *Asplenium ruprechtii* | *Asplenium x castaneoviride* (2x) | *Asplenium castaneoviride* | *Asplenium x bimixtum* |
| --- | --- | --- | --- | --- | --- |
| *Asplenium incisum* | - |  |  |  |  |
| *Asplenium ruprechtii* | 91.86 | **-** | - | - | - |
| *Asplenium* x *castaneoviride* (2x) | 91.43 | 98.5 (99.90) | - | - | - |
| *Asplenium castaneoviride* | 91.86 | 99.98 | 98.5 (99.89) | - | - |
| *Asplenium x bimixtum* | 91.84 | 99.96 | 98.48 (99.88) | 99.95 | - |
