# Supplementary material for: Dynamic hybridization between two spleenworts, Asplenium incisum and Asplenium ruprechtii in Korea
Source: Front Plant Sci. 2023 Jul 5;14:1116040. doi: 10.3389/fpls.2023.1116040 (PMC10354290; doi:10.3389/fpls.2023.1116040)
Supplement: Supplementary file 9 [file Table_5.docx]

Supplementary Table 5. Blast result of *A. ruprechtii rps2-rpoC2* region against nucleotide collection.

| Query | Subject | Subject Organism | Subject Group | Bit Score | Alignment Length | Percent Identity | E-value | Query Start | Query End | Subject Start | Subject End |
| --- | --- | --- | --- | --- | --- | --- | --- | --- | --- | --- | --- |
| *A. ruprechtii rpoC2-rps2* | HM778032.1 | Cheilanthes lindheimeri chloroplast, complete genome | Ferns | 280 | 902 | 67.184 | 4.94E-70 | 3091 | 3973 | 106092 | 106991 |
|  | KF225594.1 | Diplopterygium glaucum chloroplast, complete genome |  | 72.5 | 329 | 65.957 | 4.64E-07 | 1706 | 2028 | 4614 | 4937 |
|  | KX418656.2 | Dryopteris fragrans chloroplast, complete genome |  | 67.1 | 188 | 68.617 | 1.97E-05 | 3541 | 3724 | 13741 | 13925 |
|  | KY427332.1 | Asplenium prolongatum chloroplast, complete genome |  | 341 | 263 | 88.973 | 6.01E-88 | 3619 | 3880 | 104188 | 103926 |
|  | KY427350.1 | Hymenasplenium unilaterale chloroplast, complete genome |  | 343 | 311 | 84.566 | 4.93E-89 | 1397 | 1704 | 45327 | 45637 |
|  | KY427350.1 | Hymenasplenium unilaterale chloroplast, complete genome |  | 436 | 356 | 87.36 | 1.02E-116 | 1741 | 2096 | 44760 | 45114 |
|  | KY427356.1 | Rhachidosorus consimilis chloroplast, complete genome |  | 206 | 399 | 71.429 | 8.94E-48 | 1 | 399 | 94938 | 94540 |
|  | MH173073.1 | Cheilanthes bolborrhiza voucher DUKE:Rothfels 3294 chloroplast, partial genome |  | 655 | 645 | 82.791 | 0 | 3367 | 4008 | 11025 | 11667 |
|  | MH173073.1 | Cheilanthes bolborrhiza voucher DUKE:Rothfels 3294 chloroplast, partial genome |  | 425 | 531 | 77.589 | 1.84E-113 | 5 | 535 | 11673 | 12194 |
|  | MH173073.1 | Cheilanthes bolborrhiza voucher DUKE:Rothfels 3294 chloroplast, partial genome |  | 247 | 470 | 71.915 | 1.02E-59 | 657 | 1121 | 13744 | 14205 |
|  | MH173077.1 | Jamesonia brasiliensis voucher SP:Schuettpelz 1444 chloroplast, partial genome |  | 172 | 358 | 71.508 | 1.84E-37 | 14 | 369 | 26159 | 25804 |
|  | MH173082.1 | Pteris vittata voucher DUKE:Rothfels 4298 chloroplast, complete genome |  | 889 | 1225 | 76 | 0 | 2491 | 3701 | 28291 | 29474 |
|  | MH500228.1 | Pteris vittata chloroplast, complete genome |  | 889 | 1225 | 76 | 0 | 2491 | 3701 | 28290 | 29473 |
|  | MN599066.1 | Cyclosorus interruptus chloroplast, complete genome |  | 192 | 530 | 68.302 | 1.97E-43 | 1 | 529 | 94502 | 93974 |
|  | MN599066.1 | Cyclosorus interruptus chloroplast, complete genome |  | 303 | 859 | 67.986 | 1.51E-76 | 3151 | 4008 | 95361 | 94504 |
|  | MN623352.1 | Selliguea yakushimensis chloroplast, complete genome |  | 190 | 656 | 65.701 | 6.87E-43 | 3364 | 4003 | 117538 | 118146 |
|  | MN623352.1 | Selliguea yakushimensis chloroplast, complete genome |  | 157 | 332 | 70.783 | 1.42E-32 | 6 | 333 | 118158 | 118489 |
|  | MN623355.1 | Glaphyropteridopsis erubescens chloroplast, complete genome |  | 315 | 284 | 84.507 | 2.39E-80 | 3725 | 4008 | 105365 | 105648 |
|  | MN623355.1 | Glaphyropteridopsis erubescens chloroplast, complete genome |  | 591 | 510 | 85.686 | 2.09E-163 | 1 | 510 | 105650 | 106159 |
|  | MN623357.1 | Cyathea lepifera chloroplast, complete genome |  | 2929 | 2644 | 84.455 | 0 | 1397 | 4008 | 114976 | 117611 |
|  | MN623357.1 | Cyathea lepifera chloroplast, complete genome |  | 591 | 566 | 83.039 | 2.09E-163 | 1 | 556 | 117613 | 118170 |
|  | MN623360.1 | Oceaniopteris gibba chloroplast, complete genome |  | 966 | 1321 | 76.533 | 0 | 1592 | 2909 | 29256 | 30551 |
|  | MN623360.1 | Oceaniopteris gibba chloroplast, complete genome |  | 93.3 | 301 | 67.774 | 1.42E-13 | 1748 | 2047 | 36310 | 36014 |
|  | MN623362.1 | Plagiogyria subadnata chloroplast, complete genome |  | 104 | 120 | 79.167 | 7.85E-17 | 2 | 121 | 30960 | 30841 |
|  | MN623367.1 | Platycerium bifurcatum chloroplast, complete genome |  | 247 | 453 | 72.406 | 1.02E-59 | 41 | 492 | 93909 | 93458 |
|  | MN623367.1 | Platycerium bifurcatum chloroplast, complete genome |  | 69.8 | 220 | 67.273 | 1.62E-06 | 837 | 1056 | 91632 | 91419 |
|  | MN623367.1 | Platycerium bifurcatum chloroplast, complete genome |  | 462 | 1135 | 69.075 | 2.55E-124 | 2876 | 4008 | 95066 | 93951 |
|  | MN885667.1 | Pyrrosia petiolosa chloroplast, complete genome |  | 293 | 491 | 73.523 | 7.82E-74 | 3 | 492 | 61736 | 61247 |
|  | MN885667.1 | Pyrrosia petiolosa chloroplast, complete genome |  | 1024 | 2490 | 69.639 | 0 | 1533 | 4003 | 64183 | 61745 |
|  | MN885668.1 | Pyrrosia lingua chloroplast, complete genome |  | 297 | 491 | 73.727 | 6.42E-75 | 3 | 492 | 60635 | 60146 |
|  | MN885668.1 | Pyrrosia lingua chloroplast, complete genome |  | 412 | 795 | 71.572 | 1.16E-109 | 3210 | 4003 | 61438 | 60644 |
|  | MN885668.1 | Pyrrosia lingua chloroplast, complete genome |  | 244 | 315 | 77.46 | 3.56E-59 | 1533 | 1841 | 61751 | 61441 |
|  | MN885669.1 | Pyrrosia sheareri chloroplast, complete genome |  | 297 | 491 | 73.727 | 6.42E-75 | 3 | 492 | 60289 | 59800 |
|  | MT210539.1 | Pyrrosia lingua chloroplast, complete genome |  | 1036 | 2492 | 69.743 | 0 | 1533 | 4003 | 63046 | 60608 |
|  | MT210539.1 | Pyrrosia lingua chloroplast, complete genome |  | 364 | 492 | 77.033 | 5.27E-95 | 3 | 492 | 60599 | 60110 |
|  | MT210540.1 | Pyrrosia lingua chloroplast, complete genome |  | 1027 | 2492 | 69.663 | 0 | 1533 | 4003 | 63025 | 60587 |
|  | MT210540.1 | Pyrrosia lingua chloroplast, complete genome |  | 360 | 492 | 76.829 | 6.42E-94 | 3 | 492 | 60578 | 60089 |
|  | MT210541.1 | Pyrrosia petiolosa chloroplast, complete genome |  | 320 | 491 | 74.745 | 5.63E-82 | 3 | 492 | 61671 | 61182 |
|  | MT210541.1 | Pyrrosia petiolosa chloroplast, complete genome |  | 1021 | 2494 | 69.607 | 0 | 1533 | 4003 | 64123 | 61680 |
|  | MT210542.1 | Pyrrosia drakeana chloroplast, complete genome |  | 297 | 491 | 73.727 | 6.42E-75 | 3 | 492 | 60268 | 59779 |
|  | MT210543.1 | Pyrrosia angustissima chloroplast, complete genome |  | 1029 | 2490 | 69.679 | 0 | 1533 | 4003 | 64717 | 62279 |
|  | MT210543.1 | Pyrrosia angustissima chloroplast, complete genome |  | 293 | 491 | 73.523 | 7.82E-74 | 3 | 492 | 62270 | 61781 |
|  | MW042261.1 | Platycerium bifurcatum voucher Li 2020010101 chloroplast, complete genome |  | 1052 | 2478 | 69.935 | 0 | 1545 | 4003 | 97131 | 94700 |
|  | MW042261.1 | Platycerium bifurcatum voucher Li 2020010101 chloroplast, complete genome |  | 255 | 471 | 71.975 | 1.97E-62 | 3 | 473 | 94691 | 94221 |
|  | MW467509.1 | Platycerium wallichii chloroplast, complete genome |  | 65.3 | 127 | 72.441 | 6.88E-05 | 8 | 133 | 93600 | 93725 |
|  | MW467509.1 | Platycerium wallichii chloroplast, complete genome |  | 233 | 389 | 73.265 | 2.24E-55 | 104 | 492 | 81865 | 82249 |
|  | NC_037893.1 | Cibotium barometz chloroplast, complete genome |  | 615 | 513 | 86.55 | 1.83E-170 | 1 | 513 | 98779 | 99291 |
|  | NC_037893.1 | Cibotium barometz chloroplast, complete genome |  | 103 | 194 | 71.649 | 2.74E-16 | 2 | 195 | 98165 | 98358 |
|  | NC_038150.1 | Alsophila podophylla chloroplast, complete genome |  | 1964 | 1740 | 85.057 | 0 | 2270 | 4008 | 101111 | 99378 |
|  | NC_038150.1 | Alsophila podophylla chloroplast, complete genome |  | 1036 | 906 | 85.43 | 0 | 1397 | 2296 | 102065 | 101160 |
|  | NC_038150.1 | Alsophila podophylla chloroplast, complete genome |  | 593 | 511 | 85.714 | 5.99E-164 | 1 | 511 | 99376 | 98866 |
|  | NC_039724.1 | Myriopteris covillei voucher DUKE:Schuettpelz 443 chloroplast, complete genome |  | 260 | 843 | 67.023 | 1.61E-63 | 3152 | 3973 | 106143 | 106985 |
|  | NC_040171.1 | Pentagramma triangularis voucher DUKE:Schuettpelz 1332 chloroplast, complete genome |  | 487 | 492 | 81.911 | 6.41E-132 | 1 | 492 | 62425 | 61934 |
|  | NC_040171.1 | Pentagramma triangularis voucher DUKE:Schuettpelz 1332 chloroplast, complete genome |  | 1879 | 2376 | 77.609 | 0 | 1639 | 4008 | 64767 | 62427 |
|  | NC_040173.1 | Hemionitis subcordata voucher DUKE:Rothfels 3163 chloroplast, complete genome |  | 1129 | 1534 | 76.402 | 0 | 2546 | 4008 | 93540 | 95071 |
|  | NC_040173.1 | Hemionitis subcordata voucher DUKE:Rothfels 3163 chloroplast, complete genome |  | 904 | 1179 | 77.099 | 0 | 1398 | 2549 | 99988 | 101149 |
|  | NC_040173.1 | Hemionitis subcordata voucher DUKE:Rothfels 3163 chloroplast, complete genome |  | 434 | 526 | 78.327 | 3.55E-116 | 1 | 523 | 95073 | 95598 |
|  | NC_040173.1 | Hemionitis subcordata voucher DUKE:Rothfels 3163 chloroplast, complete genome |  | 66.2 | 164 | 69.512 | 1.97E-05 | 236 | 397 | 97572 | 97411 |
|  | NC_040203.1 | Notholaena standleyi voucher DUKE:Schuettpelz 435 chloroplast, complete genome |  | 445 | 561 | 77.54 | 1.96E-119 | 2 | 556 | 96601 | 97161 |
|  | NC_040203.1 | Notholaena standleyi voucher DUKE:Schuettpelz 435 chloroplast, complete genome |  | 1996 | 2630 | 76.92 | 0 | 1398 | 4008 | 94018 | 96598 |
|  | NC_040206.1 | Bommeria hispida voucher DUKE:Beck 1130 chloroplast, complete genome |  | 1802 | 2295 | 77.386 | 0 | 1728 | 4008 | 125529 | 123279 |
|  | NC_040206.1 | Bommeria hispida voucher DUKE:Beck 1130 chloroplast, complete genome |  | 393 | 708 | 73.023 | 1.09E-103 | 683 | 1382 | 121114 | 120418 |
|  | NC_040206.1 | Bommeria hispida voucher DUKE:Beck 1130 chloroplast, complete genome |  | 398 | 533 | 76.548 | 2.55E-105 | 3 | 535 | 123275 | 122743 |
|  | NC_040208.1 | Tryonia myriophylla voucher SP:Schuettpelz 1434 chloroplast, complete genome |  | 171 | 561 | 67.914 | 6.43E-37 | 667 | 1213 | 29026 | 28479 |
|  | NC_040208.1 | Tryonia myriophylla voucher SP:Schuettpelz 1434 chloroplast, complete genome |  | 416 | 495 | 78.586 | 9.52E-111 | 5 | 499 | 31037 | 30543 |
|  | NC_040208.1 | Tryonia myriophylla voucher SP:Schuettpelz 1434 chloroplast, complete genome |  | 1208 | 1701 | 75.661 | 0 | 2312 | 4008 | 32710 | 31043 |
|  | NC_040208.1 | Tryonia myriophylla voucher SP:Schuettpelz 1434 chloroplast, complete genome |  | 470 | 622 | 76.849 | 4.92E-127 | 1637 | 2257 | 33511 | 32890 |
|  | NC_040209.1 | Adiantum aleuticum voucher DUKE:Rothfels 4097 chloroplast, complete genome |  | 562 | 673 | 78.603 | 1.01E-154 | 3337 | 4008 | 97483 | 96811 |
|  | NC_040209.1 | Adiantum aleuticum voucher DUKE:Rothfels 4097 chloroplast, complete genome |  | 449 | 564 | 77.482 | 1.61E-120 | 6 | 569 | 96804 | 96250 |
|  | NC_040210.1 | Anogramma chaerophylla voucher SP:Prado 2178 chloroplast, complete genome |  | 195 | 327 | 73.394 | 5.64E-44 | 1 | 321 | 53383 | 53709 |
|  | NC_040212.1 | Ceratopteris cornuta voucher DUKE:Rothfels 4298 chloroplast, complete genome |  | 889 | 1225 | 76 | 0 | 2491 | 3701 | 28291 | 29474 |
|  | NC_040213.1 | Myriopteris scabra voucher DUKE:Windham 3495 chloroplast, complete genome |  | 169 | 515 | 67.767 | 2.25E-36 | 1 | 495 | 95370 | 95882 |
|  | NC_040214.1 | Calciphilopteris ludens voucher DUKE:Huiet s.n. chloroplast, complete genome |  | 402 | 564 | 76.064 | 2.10E-106 | 1 | 563 | 93924 | 94486 |
|  | NC_040214.1 | Calciphilopteris ludens voucher DUKE:Huiet s.n. chloroplast, complete genome |  | 341 | 433 | 77.598 | 6.01E-88 | 3572 | 4003 | 93485 | 93917 |
|  | NC_040215.1 | Haplopteris elongata voucher BO:Schuettpelz 1559 chloroplast, complete genome |  | 239 | 744 | 68.011 | 1.51E-57 | 3271 | 4008 | 95026 | 94288 |
|  | NC_040215.1 | Haplopteris elongata voucher BO:Schuettpelz 1559 chloroplast, complete genome |  | 195 | 497 | 69.215 | 5.64E-44 | 5 | 498 | 94282 | 93789 |
|  | NC_040220.1 | Histiopteris incisa chloroplast, complete genome |  | 358 | 863 | 69.757 | 2.24E-93 | 3154 | 4008 | 96194 | 97049 |
|  | NC_040220.1 | Histiopteris incisa chloroplast, complete genome |  | 197 | 469 | 69.723 | 4.63E-45 | 56 | 520 | 97106 | 97573 |
|  | NC_040226.1 | Pyrrosia bonii chloroplast, complete genome |  | 297 | 491 | 73.727 | 6.42E-75 | 3 | 492 | 60253 | 59764 |
|  | NC_041428.1 | Mesopteris tonkinensis chloroplast, complete genome |  | 518 | 457 | 85.12 | 1.08E-141 | 1388 | 1844 | 89712 | 90168 |
|  | NC_041428.1 | Mesopteris tonkinensis chloroplast, complete genome |  | 335 | 299 | 85.284 | 2.56E-86 | 1100 | 1396 | 88741 | 89033 |
|  | NC_041428.1 | Mesopteris tonkinensis chloroplast, complete genome |  | 2398 | 2203 | 84.067 | 0 | 1843 | 4008 | 85252 | 87453 |
|  | NC_041428.1 | Mesopteris tonkinensis chloroplast, complete genome |  | 644 | 529 | 86.957 | 3.78E-179 | 1 | 529 | 87455 | 87983 |
|  | NC_044079.1 | Alsophila gigantea chloroplast, complete genome |  | 997 | 918 | 84.205 | 0 | 1388 | 2296 | 70402 | 69485 |
|  | NC_044079.1 | Alsophila gigantea chloroplast, complete genome |  | 603 | 539 | 84.787 | 3.31E-167 | 1 | 539 | 67683 | 67145 |
|  | NC_044079.1 | Alsophila gigantea chloroplast, complete genome |  | 1976 | 1743 | 85.198 | 0 | 2270 | 4008 | 69421 | 67685 |
|  | NC_044685.1 | Pecluma dulcis voucher TUR:Jones 919 chloroplast, complete genome |  | 86.9 | 240 | 68.75 | 2.11E-11 | 1790 | 2028 | 56462 | 56697 |
|  | NC_044687.1 | Tectaria panamensis voucher TUR:Jones 1052 chloroplast, complete genome |  | 329 | 617 | 72.285 | 1.09E-84 | 683 | 1290 | 96814 | 97429 |
|  | NC_044687.1 | Tectaria panamensis voucher TUR:Jones 1052 chloroplast, complete genome |  | 92.4 | 93 | 81.72 | 4.95E-13 | 1398 | 1490 | 93802 | 93894 |
|  | NC_044687.1 | Tectaria panamensis voucher TUR:Jones 1052 chloroplast, complete genome |  | 409 | 555 | 76.396 | 1.41E-108 | 5 | 556 | 94643 | 95197 |
|  | NC_044687.1 | Tectaria panamensis voucher TUR:Jones 1052 chloroplast, complete genome |  | 604 | 711 | 79.044 | 3.31E-167 | 3300 | 4008 | 93927 | 94637 |
|  | NC_044688.1 | Davallia solida var. fejeensis voucher TUR:HBG_0044-0736 chloroplast, complete genome |  | 262 | 485 | 71.959 | 1.33E-64 | 8 | 492 | 106361 | 106845 |
|  | NC_044688.1 | Davallia solida var. fejeensis voucher TUR:HBG_0044-0736 chloroplast, complete genome |  | 848 | 2117 | 69.343 | 0 | 1907 | 4003 | 104277 | 106347 |
|  | NC_045119.1 | Asplenium nidus chloroplast, complete genome |  | 562 | 535 | 83.364 | 1.01E-154 | 2 | 536 | 12901 | 12369 |
|  | NC_045119.1 | Asplenium nidus chloroplast, complete genome |  | 65.3 | 105 | 74.286 | 6.88E-05 | 29 | 133 | 12350 | 12247 |
|  | NC_045119.1 | Asplenium nidus chloroplast, complete genome |  | 2731 | 2657 | 82.65 | 0 | 1391 | 4008 | 15545 | 12904 |
|  | NC_046784.1 | Plagiogyria euphlebia chloroplast, complete genome |  | 96.9 | 284 | 67.958 | 1.16E-14 | 1748 | 2028 | 33091 | 32808 |
|  | NC_046784.1 | Plagiogyria euphlebia chloroplast, complete genome |  | 109 | 215 | 71.163 | 1.85E-18 | 2 | 216 | 30825 | 30611 |
|  | NC_047436.1 | Pyrrosia subfurfuracea chloroplast, complete genome |  | 293 | 491 | 73.523 | 7.82E-74 | 3 | 492 | 60291 | 59802 |
|  | NC_053768.1 | Acrostichum speciosum chloroplast, complete genome |  | 77.9 | 252 | 68.254 | 1.09E-08 | 8 | 256 | 47487 | 47735 |
|  | NC_053768.1 | Acrostichum speciosum chloroplast, complete genome |  | 91.5 | 466 | 64.592 | 4.95E-13 | 28 | 472 | 96326 | 96789 |
|  | FO905834.1 | Leptosphaeria biglobosa Thlaspii ibcn65_scaffold00067 complete sequence | Fungi | 60.8 | 50 | 88 | 0.000838 | 509 | 557 | 39551 | 39600 |
